# Surgical stabilization of spinal metastasis in diffuse idiopathic skeletal hyperostosis (“Mets-on-DISH”): Two case reports

**DOI:** 10.1097/MD.0000000000020397

**Published:** 2020-05-29

**Authors:** Atsuyuki Kawabata, Takashi Hirai, Ryo Tohara, Masato Yuasa, Hiroyuki Inose, Hirotaka Koyanagi, Shingo Sato, Kurando Utagawa, Jun Hashimoto, Atsushi Okawa, Toshitaka Yoshii

**Affiliations:** a Department of Orthopedic Surgery, Tokyo Medical and Dental University, Tokyo, Japan; b Department of Orthopedic Surgery, Tsuchiura Kyodo General Hospital, Tsuchiura City, Ibaraki, Japan.

**Keywords:** diffuse idiopathic skeletal hyperostosis, instrumentation, metastasis, minimally invasive surgery, spinal instability

## Abstract

**Rationale:**

Diffuse idiopathic skeletal hyperostosis (DISH) is characterized by ossification along the anterolateral aspect of at least 4 contiguous vertebral bodies. A fracture involving the fused vertebra in patients with DISH often leads to severe instability and spinal cord injury. Spinal metastasis (Mets) and DISH can coexist in elderly patients and increase their risk of pathologic vertebral fractures. However, there are few reports on concomitant spinal Mets and DISH.

**Patient concerns:**

A 78-year-old man who complained of gradual onset of paraparesis, sensory loss below the umbilicus, and incontinence (case 1) and a 63-year-old woman who complained of severe back pain and urinary incontinence (case 2).

**Diagnosis:**

Two patients were diagnosed with spinal Mets and DISH.

**Interventions:**

Decompression surgery was performed at the metastatic sites in case 1 whereas instrumentation surgery was performed in case 2 despite the fracture having a benign appearance with no associated neurologic symptoms.

**Outcomes:**

A vertebral fracture developed at the metastatic vertebra after decompression surgery in case 1. Severe instability of the surgical site in this case resulted in persistent paralysis even after subsequent revision surgery with instrumentation. In contrast, the clinical course was benign without any neurologic dysfunction at the 2-year follow-up in case 2.

**Lessons:**

Instrumentation surgery should be performed in patients with DISH who develop spinal Mets even if there is no apparent instability.

## 1 Introduction

The increasing aging of society is now presenting surgical problems that have rarely been encountered before. Cancer and diffuse idiopathic skeletal hyperostosis (DISH) are both conditions typically found in the older population, and pathologic fracture due to spinal metastasis (Mets) inside an area of DISH may be more common than before.^[1]^ However, little is known about the relationship between metastatic cancer to the spine and DISH in terms of treatment and outcomes. We have encountered 2 patients with spinal Mets associated with DISH, 1 of whom underwent decompression surgery alone while the other underwent instrumentation surgery without decompression.

## 2 Statement of informed consent

Informed consent for publication of the data was obtained from both patients prior to participating in the study.

## 3 Case presentations

### 3.1 Case 1

A 78-year-old man presented to our outpatient department with a history of gradual onset of paraparesis, sensory loss below the umbilicus, and incontinence. He also had mild back pain. He had a history of prostate cancer with intra-pelvic lymph node Mets. The TNM classification was cT2aN1M1b. He had undergone tumor resection surgery 2 years earlier and subsequently received chemoradiotherapy. His medical history was unremarkable apart from the prostatic cancer.

**Figure 1:**
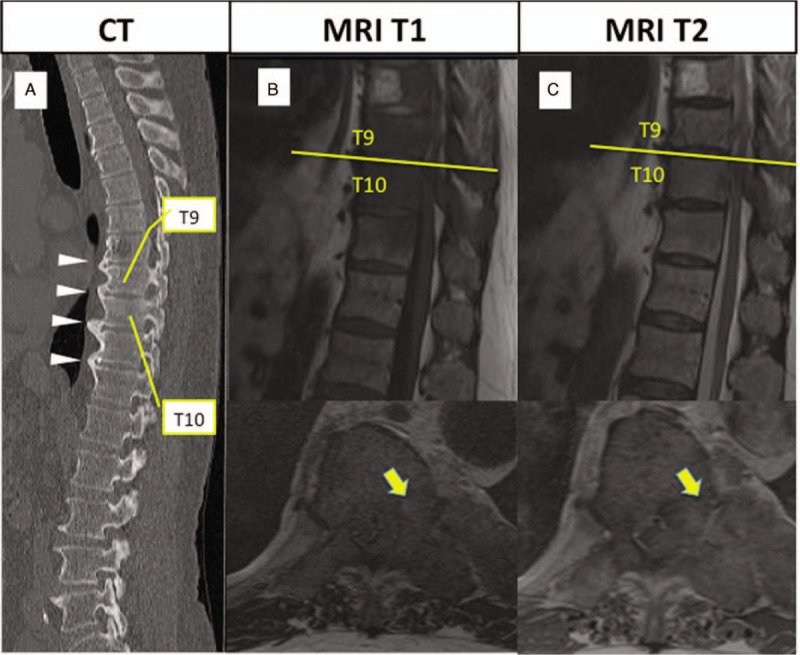
(A) CT scan showing fused vertebra from T8 to T12 because of ossification of anterior longitudinal ligament (white arrow). (B) T1-weighted and (C) T2-weighted magnetic resonance imaging scans showing spinal metastasis in the vertebra at T9 and T10 and left pedicle of T10 with rib metastasis (yellow arrow).

His vital signs were normal at the first visit. He was alert and oriented but had difficulty standing up independently. Physical examination revealed incomplete sensory loss below T10 on both sides. Muscle function was impaired in both lower extremities (power 2/2 on the Medical Research Council scale).^[2]^ Computed tomography (CT) revealed fusion of T8 to T12 due to ossification of the anterior longitudinal ligament (Fig. 1A) and Mets from the left ninth to eleventh rib. Magnetic resonance imaging (MRI) revealed spinal Mets at the T10 vertebra but no fracture (Fig. 1B, C). The metastatic site was more osteoplastic than osteolytic. The patient had a Spinal Instability Neoplastic Score^[3]^ of 4 and an American Spinal Injury Association grade of C. His revised Tokuhashi Score^[4]^ was 8 with unresectable lung Mets on admission. Given that the site appeared to be mechanically stable because of the fused vertebra, we performed decompression surgery from T9 to T10, including left pediculectomy, without instrumentation.

A bone-modifying agent (denosumab) was started postoperatively and a course of radiation therapy was administered. After intensive rehabilitation, his paralysis gradually improved to the point that he was able to walk with the aid of a T-cane.

**Figure 2:**
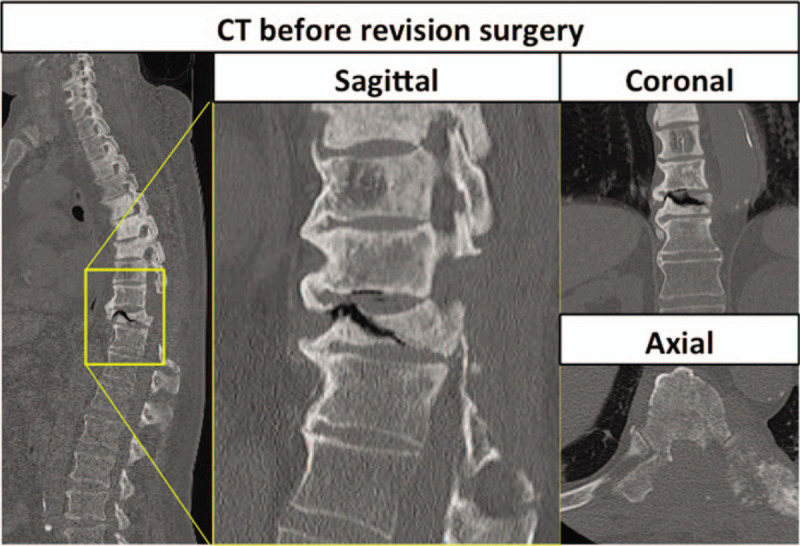
Preoperative computed tomography scan showing fracture of the vertebra at T10 with an accompanying intra-vertebral vacuum cleft sign.

**Figure 3:**
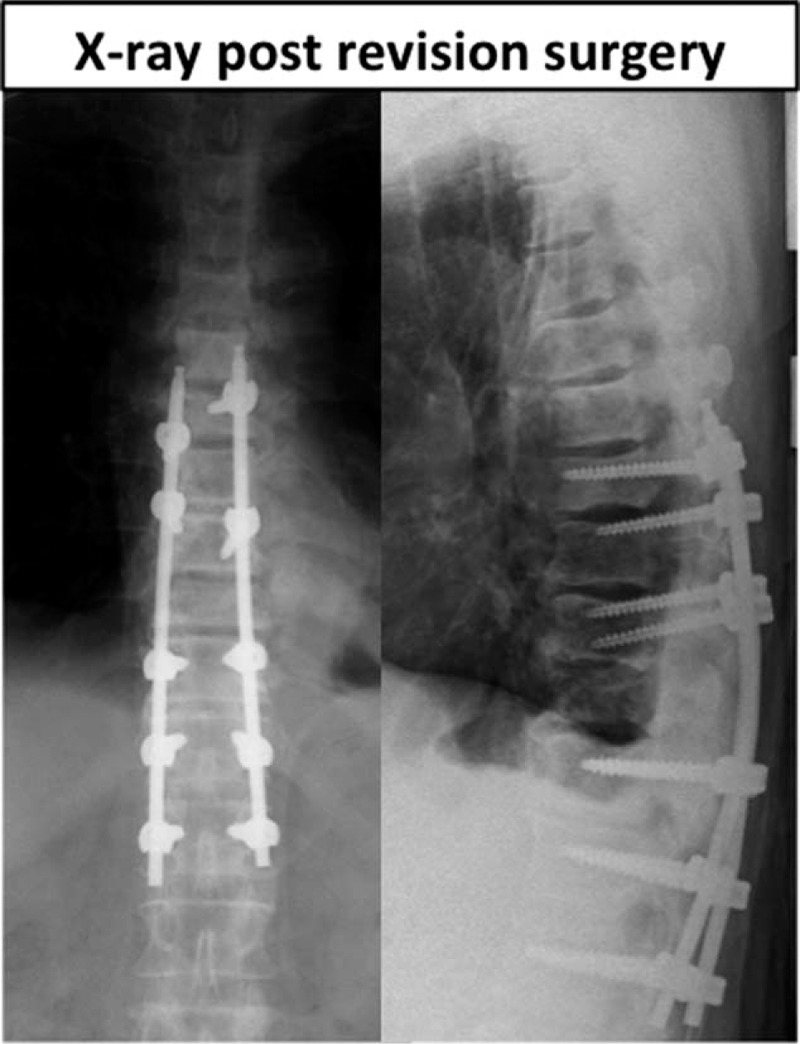
Radiographs obtained after posterior fixation surgery from T7 to L1.

Seven months after the surgery, he had a recurrence of motor weakness and was readmitted to our hospital when he became unable to walk. Physical examination revealed complete loss of pinprick sensation below the T10 level and impaired muscle function in both lower extremities (power 2/2 on the Medical Research Council scale). CT and MRI revealed a pathological fracture of the T10 vertebra with a transverse fracture line. CT also showed an intravertebral vacuum cleft sign at T10, indicating severe spinal instability (Fig. 2). We diagnosed spinal cord injury due to instability and performed posterior fixation surgery from T7 to L1 on the day of admission (Fig. 3). There was no recovery of neurologic function postoperatively, and the patient succumbed to lung Mets 3 months after revision surgery.

### 3.2 Case 2

The patient was a 63-year-old woman who complained of severe back pain and urinary incontinence. She had undergone a total mastectomy and sentinel lymph node biopsy for right breast cancer 3 years earlier. Her past medical history was significant for diabetes, hypertension, asthma, and Graves disease. Multiple bone metastases involving the vertebral body at T8, the eighth and tenth ribs, and both sides of the ilium were detected on bone scintigraphy. Therefore, the patient was referred to our hospital as a potential candidate for surgery. She had been taking denosumab for 3 years and had received radiation therapy but continued to have persistent back pain and urinary incontinence.

On examination, she was alert and oriented and her vital signs were within normal limits. Physical examination showed no motor weakness or sensory loss. However, she could not maintain a seated position because of severe back pain.

**Figure 4:**
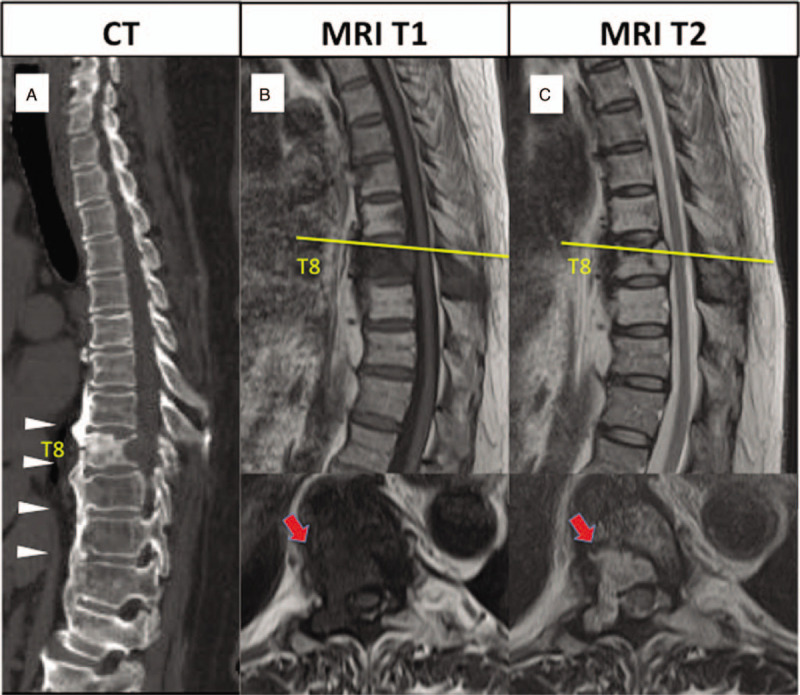
(A) Computed tomography scan reveals fusion of vertebra from T5 to T12 because of ossification of the anterior longitudinal ligament (white arrow). (B) T1-weighted and (C) T2-weighted MRI scans show spinal Metastasis at the T8 vertebra and the right pedicle with no stenosis.

**Figure 5:**
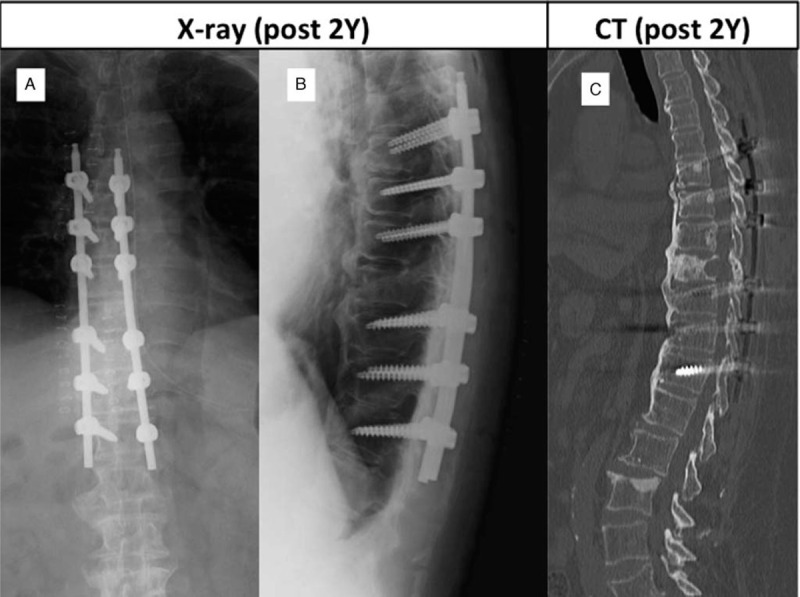
(A, B) Radiographs and (C) computed tomography scan showing ossification and fusion of the site of Metastasis at T8.

Lumbosacral radiographs revealed only the expected degenerative changes. CT revealed fused vertebra at T5 to T12 due to ossification of the anterior longitudinal ligament, which was diagnosed as DISH (Fig. 4A). MRI showed spinal Mets at the T8 vertebra and the right pedicle, but the height of the vertebra was well preserved (Fig. 4B, C). There was no stenosis in the spinal canal. The patient had a Spinal Instability Neoplastic Score of 5 and an American Spinal Injury Association grade of E. Her Tokuhashi Score was 13 on admission. We performed posterior fusion without decompression using a minimally invasive technique from T6 to T12 to stabilize the ankylosing thoracic spine. Her back pain improved soon after the surgery and she could walk without a cane. At the 2-year follow-up visit, radiographs (Fig. 5A, B) and CT scans (Fig. 5C) showed ossification and fusion of the T8 vertebra with adjacent segments but no complications. Positron emission tomography was performed on several occasions during follow-up and showed a decrease in the standardized uptake value at the T8 vertebra.

## 4 Discussion

The number of patients diagnosed with cancer is increasing because of the rapid aging of society. Although various treatments for cancer are being steadily developed and the life expectancy of cancer patients may be increasing, the frequency of skeletal-related events due to bone Mets is thought to be increasing.^[5]^ Bone metastases often arise in the spinal column and cause pathologic fractures, which lead to severe back pain and even nerve damage. These fractures represent significant pathology that diminishes a patient's quality of life, so early diagnosis and appropriate treatment is mandatory. The treatments available can be surgical or nonsurgical. Nonsurgical treatments include chemotherapy, radiotherapy, bone-modifying agents, and several types of corset. Although these methods are less invasive than surgery, they cannot stabilize an unstable spinal column. Surgery with instrumentation seems to achieve strong stabilization of the spine,^[6]^ which prevents further vertebral instability. However, surgery has several disadvantages, including invasiveness and a risk of operative complications, such as nerve damage or surgical site infection. Furthermore, 1 study reported that surgery was no more effective than radiotherapy alone for metastatic spinal cord compression.^[7]^ Therefore, decision-making about which treatment is appropriate is generally made by attending surgeons according to their experience and consideration of the patient's overall health status, likely prognosis, and local stability of the spine with metastatic disease.

DISH is a pathologic state characterized by bony proliferation at the sites of tendinous and ligamentous insertion of the anterior longitudinal ligament in the spine. The diagnostic criteria most commonly used were devised by Resnick and Niwayama.^[8]^ These criteria include ossification along the anterolateral aspect of at least 4 contiguous vertebral bodies. The condition is usually asymptomatic and is often an incidental finding on imaging performed for other reasons.^[9]^ DISH causes ankylosis of the affected area of the spine, rendering it prone to fracture even by minor trauma. The stiffness produced by the fused spinal column causes larger lever arms and stress at the fracture site, such that even fractures that appear to be benign can cause severe instability of the spine with a high rate of immediate or delayed neurologic deficit.^[10–12]^ Okada et al reported that 84% of DISH-related fractures needed surgery. Furthermore, the diagnosis of a DISH-related fracture was reported to be delayed in 19% of cases,^[10]^ mostly because of delay on the part of the attending doctor. This finding suggests that even some orthopedists are unaware that fractures associated with DISH cause spinal instability and put patients at increased risk of spinal cord injury.

The peak occurrence of DISH is in patients in their 60s.^[13,14]^ Cancer and DISH can coexist in older patients, and might cause pathologic fractures inside the area of DISH (Mets-on-DISH), which can increase the risk of structural instability of the spine. However, poor general health in patients with cancer may cause surgeons to hesitate before offering surgical treatment. Thus far, there has been no research on the relationship between metastatic cancer and DISH in terms of treatment and outcomes.

We encountered 2 cases of spinal Mets associated with DISH, each of which had different clinical courses. Decompression surgery at sites of Mets was performed alone in 1 case because there was no fracture or instability. Furthermore, we anticipated that the vertebra fused in DISH might work as a structural support. However, the outcome was a vertebral fracture and severe instability at the surgical site. This finding explains how fenestration of the posterior structure in DISH might lead to critical instability at the site. In our other case, instrumentation surgery resulted in a benign clinical course without any neurologic dysfunction at the 2-year follow-up, at which time the vertebral fracture was healed with a reduced standardized uptake value on positron emission tomography. The different outcomes in these 2 patients indicate that surgical instrumentation should be performed in patients with Mets-on-DISH, even if there is no fracture or the fracture appears stable and has a benign appearance.

## 5 Conclusion

In this report, we have described the different clinical courses in 2 cases of spinal Mets associated with DISH. We found that a correct diagnosis is needed and that surgical instrumentation should be considered even if there is no fracture or if the fracture is stable.

## Acknowledgments

I am particularly grateful for the assistance given by Ms. Yukiko Oya and Nobuko Nakajima.

## Author contributions

**Conceptualization:** Takashi Hirai, Hirotaka Koyanagi, Shingo Sato.

**Data curation:** Ryo Tohara.

**Investigation:** Masato Yuasa, Hirotaka Koyanagi, Shingo Sato, Kurando Utagawa, Jun Hashimoto.

**Supervision:** Takashi Hirai, Hiroyuki Inose, Atsushi Okawa, Toshitaka Yoshii.

**Writing – original draft:** Atsuyuki Kawabata, Ryo Tohara.

**Writing – review & editing:** Takashi Hirai.
